# A rare case of West Nile virus leading to Acute Transverse Myelitis

**DOI:** 10.51894/001c.144611

**Published:** 2025-09-30

**Authors:** Astha Nepal, Syed Hussaini

**Affiliations:** 1 Department of Family Medicine McLaren Flint

## 105

### INTRODUCTION

Transverse myelitis is an inflammatory condition of the spinal cord with multifactorial causes. Late-onset post-infectious transverse myelitis due to WNV is rare. West Nile virus (WNV) is an arbovirus, with only 1% presenting as a neuroinvasive disease.

### CASE DESCRIPTION

46-year-old female living in Michigan presented to the hospital with progressively ascending paresthesia and numbness of the bilateral lower extremities and right arm with difficulty in walking, saddle paresthesia, and difficulty in emptying the bladder. Symptoms were acute on onset, started a few hours before presenting to the hospital, and gradually progressed with peak symptoms by 48 hours. The patient denied fever, recent infections, vaccinations, trauma, recent travel, night sweats, chemical/substance/radiation exposure or past sexually transmitted infections. Neurological examination was significant for mild paraparesis, absent joint position sensation at the great toes and subjective numbness involving all four limbs. Visual field testing was intact. Blood work showed no leukocytosis. Brain MRI with and without contrast was inconclusive. MRI of the thoracic spine and lumbar spine was unremarkable. CT scan of the head was negative for brain stroke/hemorrhage. A pelvic ultrasound showed a right ovarian cyst but no signs of malignancy. Hemoglobin level, Vitamin B12, and folate level were normal ruling out anemia. Laboratories results (i.e., TSH, ESR, CRP, ANA, anti-SSA, and anti-SSB) rule out any autoimmune cause of Transverse Myelitis. Anti-GQ1B negative with no ocular involvement ruled out Guillain-Barre syndrome, Miller-Fisher syndrome, and Bickerstaff Brainstem encephalitis. Cerebrospinal fluid (CSF) analysis showed mild pleocytosis (12 WBCs with 64% polymorphs), elevated total protein, and normal glucose with WNV serology negative for IgM but positive for WNV IgG. Patient was unresponsive to high dose steroid, then started on intravenous immunoglobin and transferred to inpatient rehab.

### DISCUSSION/CONCLUSION

Michigan is endemic to WNV with around 31 cases of WNV identified in 2024. With a 1% prevalence of neuroinvasive West Nile Virus, there is only a 0.31% chance of getting neuroinvasive West Nile Virus in Michigan. Most post-infectious transverse myelitis occurs soon after the infection, but our patient presented with WNV neuroinvasive conditions at least after 90 days of infection with WNV IgM negative.

